# Cross-Coupled Sliding Mode Synchronous Control for a Double Lifting Point Hydraulic Hoist

**DOI:** 10.3390/s23239387

**Published:** 2023-11-24

**Authors:** Chungeng Sun, Xiangxiang Dong, Jipeng Li

**Affiliations:** Faculty of Mechanical and Electrical Engineering, Kunming University of Science and Technology, Kunming 650500, China; 17600132502@163.com (X.D.); ljpkust@163.com (J.L.)

**Keywords:** double lifting point hydraulic hoist, sliding mode control, cross-coupled synchronization control, electro-hydraulic servo system

## Abstract

This paper proposes a sliding mode synchronous control approach to enhance the position synchronization performance and anti-interference capability of a double lifting point hydraulic hoist. Building upon the cross-coupling synchronous control method, a coupling sliding mode surface is formulated, incorporating the single-cylinder following error and double-cylinder synchronization error. Additionally, a sliding mode synchronous controller is devised to ensure the convergence of both the single-cylinder following and synchronization error. The hyperbolic tangent function is introduced to reduce the single-cylinder following error and the buffeting of the double-cylinder synchronization error curve under sliding mode synchronous control. The simulation results show that the synchronization accuracy of the sliding mode cross-coupling synchronization control in the initial stage of the system is 53.1% higher than that of the Proportional-Derivative (PD) cross-coupling synchronization, and the synchronization accuracy in the steady state of the system is improved by 90%. The designed synchronous controller has better performance under external disturbances.

## 1. Introduction

In recent years, due to the irreplaceable advantages of hydraulic hoists and the rapid development of hydraulic technology, especially with the advancement of modern control theory and computer technology, hydraulic hoists have been increasingly used in water conservancy projects. The 2 × 6300 KN low-hole arc working gate hydraulic hoist produced by Bosch Rexroth is currently the largest arc working gate hydraulic hoist in the world. To ensure that accidents such as gate tilting do not occur during the operation of the double lifting point gate hoist, researching the synchronous control of the double hydraulic cylinders is necessary.

The performance of the single-cylinder controller plays a vital role in the performance of the synchronous controller. The electro-hydraulic servo system has nonlinear characteristics, and the traditional Proportional-Integral-Derivative (PID) control is always unsatisfactory. Feng et al. [1] designed and used an improved Particle Swarm Optimization (PSO) strategy to optimize the parameters of the PID controller, thereby improving the tracking accuracy of the electro-hydraulic position servo system. Deng et al. [2] proposed a controller suitable for the electro-hydraulic servo systems that does not require speed measurement and estimated the unmeasurable speed signal by establishing an extended state observer. This control strategy bridges the gap between disturbance observer-based and adaptive control, breaking through their limitations in practical systems. To weaken the influence of parameter uncertainty and uncertain nonlinearity in electro-hydraulic servo systems, scholars have proposed methods such as state observation [3] and automatic nonlinear control strategies such as disturbance rejection control [4,5,6,7]. Wang et al. [8] designed a series controller that combines active disturbance rejection control (ADRC) with dead zone anti-compensation to achieve effective compensation for the dead area of the proportional valve in the electro-hydraulic position servo system, thus improving the electro-hydraulic proportional valve’s features of the dead zone, the dynamic characteristics of the system, and the position tracking accuracy. Jin et al. [9] proposed a new linear active disturbance rejection control (LADRC) method that effectively suppresses interference in electro-hydraulic servo systems. Compared with the fractional-order integral derivative (FOID) control strategy proposed in the literature [10], sliding mode control (SMC) has gained increasing attention when there are external disturbances and parameter variations in the system, owing to its invariance advantages [11,12]. Cheng et al. [13] introduced a new second-order SMC design method based on the combination of a fractional-order proportional–integral–differential sliding mode surface and a state observer, and this method reduced steady-state error by introducing an integral term on the sliding mode surface, ultimately reducing system chattering and improving control accuracy. Feng et al. [14] introduced a novel adaptive sliding mode control method, SMC-RBF, which utilized an Radial-Basis Function (RBF) neural network to approximate and compensate for load interference and modeling uncertainty in the electro-hydraulic servo system. To ensure system stability, an adaptive mechanism that could adjust the connection weights of the RBF neural network was designed, nonlinear terms were introduced into the sliding mode, and an adaptive terminal SMC structure was created. As a result, the proposed SMC-RBF controller exhibited superior tracking performance and interference immunity compared to the PID controller.

The hydraulic synchronous control system is evolving and improving thanks to the tireless work of several academics and professionals [15,16,17,18,19]. Currently, three typical closed-loop synchronous control methods are parallel synchronous control, master–slave synchronous control, and cross-coupled synchronous control. The characteristic of parallel synchronous control technology is that each actuator in two channels synchronizes the feedback signal separately and records the motion error in a parallel structure [20,21,22], resulting in no coupling connection between the two channels. Rehman et al. [23] employed a feed-forward controller and a fuzzy position tracker within the decoupling controller of a single hydraulic cylinder to enhance position-tracking accuracy. Additionally, a fuzzy force tracker was utilized in the coupling controller of double hydraulic cylinders to bolster interference resistance in parallel synchronous control. A fuzzy PID control-based synchronization control technique was presented by Zhang et al. [24]. The good synchronization precision of double hydraulic cylinders under this control was obtained by the co-simulation of AMESim and Simulink. In the literature [25], the hydraulic system was modeled and adjusted through a fuzzy PID controller optimized by particle swarm optimization; the simulation showed that the fuzzy PID with particle swarm optimization was more accurate for hydraulic system synchronization control. In the literature [26], an adaptive FxLMS algorithm was used in the synchronous control of a dual-shaker system; considering the dynamic coupling between the shakers, the simulation was presented to verify the effectiveness of the control algorithm. The master–slave synchronous control strategy, as described in references [27,28,29], employs a series structure in which the slave hydraulic circuit initially follows the output signal of the main hydraulic circuit instead of tracking the desired signal from the outset [30]. A master–slave PID synchronous controller was designed by combining PID control with master–slave synchronous control. This controller incorporated proportional valve dead band compensation, and experimental results demonstrated its suitability for tunnel boring applications. In the literature [31], a fuzzy control method was introduced to eliminate the tracking error of hydraulic cylinders and the synchronization error between hydraulic cylinders. There was no coupling between the two channels in both master–slave and parallel synchronous control with the load imbalance of double hydraulic cylinders. Consequently, the controllers in both modes were relatively simple and exhibited a reasonably general synchronization accuracy. Under cross-coupling control (CCC) [32,33], a coupling relationship exists between the two channels, leading to the detection of output deviations in the executive components of both channels. This detected feedback is then fed back to the controller. Cross-coupling control combines the advantages of both parallel synchronous control and master–slave synchronous control, resulting in improved synchronization accuracy. In the literature [34], a cross-coupling control scheme was proposed, which combined two independent single hydraulic cylinder feed-forward controllers with a fuzzy synchronization error coordination controller, all acting on double hydraulic cylinders. Experimental results demonstrated the effectiveness of this approach in achieving position synchronization in an electro-hydraulic system with double hydraulic cylinders. Meanwhile, in the literature [35], a decoupling controller was utilized to compensate for input and load disturbances through feedback. This method combined a fuzzy PID controller with decoupling control based on cross-coupling to achieve synchronous control of double hydraulic cylinders. Experimental results highlighted the enhanced anti-interference capability and robustness of the synchronous system achieved through this approach. In order to address the synchronization error brought on by the uneven stress of the hydraulic bending machine’s double cylinders during operation, a single-neuron PID cross-coupling control approach was presented by Yang et al. [36]. Zhang et al. [37] proposed an adaptive sliding mode control (ASMC) for an electro-hydraulic shaking tables system. The proposed ASMC was then introduced to CCC to improve the synchronization control performance. The test results indicate that the proposed ASMC has admirable dynamic performance, exact control accuracy, and reliable, robust stability.

The aforementioned study indicates that the use of closed-loop control is often employed inside hydraulic synchronization control systems to enhance the precision of those systems. The synchronization precision and stability of the hydraulic synchronous control system may be greatly increased by the controller by outputting the signal adjustment amount to compensate for the hydraulic system’s synchronization error [38,39,40,41].

In this study, based on the cross-coupling synchronous control mode, a synchronous controller is designed using the hyperbolic tangent function. This controller simultaneously addresses the single-cylinder following error and the double-cylinder synchronization error, enabling the double-cylinder to rapidly track the desired trajectory and reduce synchronization errors with strong robustness. Co-simulation using AMESim/Simulink is conducted to compare the designed sliding mode synchronous controller with a PD-coupled synchronous controller, thus verifying the validity of the proposed method. The contributions of this article are summarized as follows. First, the adaptive approach rate was designed, including single-cylinder following error and double-cylinder synchronization error. Second, compared with PD cross-coupling control that does not rely on specific modeling among the coupling control items, sliding mode synchronous control that relies on specific modeling was applied. Finally, the coupling sliding mode surface designed in this paper has a certain degree of novelty.

## 2. Problem Formulation and Dynamic Models

The paper uses the four-way, three-position (4/3) valve-controlled asymmetric hydraulic cylinder as the power mechanism for the electro-hydraulic servo system. The mathematical model of the single-cylinder electro-hydraulic servo system is investigated. The relationship between the servo valve’s bandwidth and the power mechanism’s natural frequency dictates the form of the servo valve’s dynamic equation. When the bandwidth of the servo valve significantly exceeds the frequency of the power mechanism, the dynamics of the servo valve to that of a proportional link can be simplified. At this time, the relationship between the control input quantity u and the spool displacement $x_{v}$ is

(1) $$x_{v}=\tau u,$$

where τ is the electro-hydraulic servo valve spool displacement–control signal proportional coefficient, which is a positive number.

The flow rate flowing into the electro-hydraulic servo valve q is proportional to the control signal u input to the servo valve, and the output flow is

(2) $$q=C_{d}A\sqrt{\frac{2}{\rho }\Delta p}=C_{d}\omega x_{v}\sqrt{\frac{2}{\rho }\Delta p}=C_{d}\omega \tau u\sqrt{\frac{2}{\rho }\Delta p},$$

where q is the output flow of the servo valve, $C_{d}$ is the flow coefficient, A is the opening amount of the throttling edge, ρ is the density of the oil, Δp is the actual pressure drop before and after the throttling edge, ω is the area gradient of the servo valve port, and u is the actual control input of the servo valve (V).

According to Equations (2) and (3), the expression of the rated flow of the servo valve is provided by

(3) $$q_{N}=C_{d}\omega \tau u_{\max}\sqrt{\frac{2}{\rho }\Delta p_{N}},$$

where $u_{\max}$ is the maximum control signal of the servo valve, $q_{N}$ is the rated flow rate of the valve corresponding to $u_{\max}$, and $\Delta p_{N}$ is the rated pressure drop of the servo valve.

According to Equation (3), the flow equation of the electro-hydraulic servo valve is obtained as follows:

(4) $$q_{1}=q_{N}\frac{u}{u_{\max}}\sqrt{\frac{\Delta p_{1}}{\Delta p_{N}}},$$

(5) $$q_{2}=q_{N}\frac{u}{u_{\max}}\sqrt{\frac{\Delta p_{2}}{\Delta p_{N}}},$$

(6) $$\Delta p_{1}=s\left(u\right)\left(p_{s}-p_{1}\right)+s\left(-u\right)\left(p_{1}-p_{0}\right),$$

(7) $$\Delta p_{2}=s\left(u\right)\left(p_{2}-p_{0}\right)+s\left(-u\right)\left(p_{s}-p_{2}\right).$$

The function is defined by the following:

(8) $$s(*)=\left\{\begin{array}{l} 1\left(*\geq 0\right)\\ 0\left(*<0\right) \end{array}\right.,$$

where u is the control input of the servo valve, $\Delta p_{1}$ is the actual pressure drop on the left side of the servo valve, and $\Delta p_{2}$ is the actual pressure drop on the right side of the servo valve. $p_{0}$ is the return pressure of the system, $p_{s}$ is the supply pressure of the system, and $p_{1}$ and $p_{2}$ are the piston-side chamber pressure and the rod-side chamber pressure of the hydraulic cylinder, respectively.

With the advancement of hydraulic sealing technology, compared with the internal leakage of the hydraulic cylinder, external leakage is very minor and can be ignored. Therefore, only internal leakage needs to be considered when modeling. The hydraulic cylinder flow continuity equation is presented in Equations (9)–(12):

(9) $$q_{1}=A_{1}\frac{dx_{p}}{dt}+C_{t}\left(p_{1}-p_{2}\right)+\frac{V_{1}}{\beta_{e}}\frac{dp_{1}}{dt},$$

(10) $$q_{2}=A_{2}\frac{dx_{p}}{dt}+C_{t}\left(p_{1}-p_{2}\right)-\frac{V_{2}}{\beta_{e}}\frac{dp_{2}}{dt},$$

(11) $$V_{1}=V_{01}+A_{1}x_{p},$$

(12) $$V_{2}=V_{02}+A_{2}\left(y-x_{p}\right),$$

where $q_{1}$ is the flow rate of the piston-side chamber of the hydraulic cylinder; $q_{2}$ is the flow rate of the rod-side chamber of the hydraulic cylinder; $\beta_{e}$ is the oil bulk elastic modulus; $C_{t}$ is the leakage coefficient in the hydraulic cylinder; $V_{1}$ is the total volume in the piston-side chamber of the hydraulic cylinder (m^3^); $V_{2}$ is the total volume in the rod-side chamber of the hydraulic cylinder; $V_{01}$ is the total volume in the piston-side chamber of the hydraulic cylinder (m^3^); $V_{02}$ is the initial volume in the rod-side chamber of the hydraulic cylinder (m^3^); y is the displacement of the hydraulic cylinder (m); $A_{1}$ and $A_{2}$ are the effective area of the piston of the hydraulic cylinder and the effective area of the rod-side; $x_{p}$ is the displacement of the piston.

To intuitively reflect the state of the system, Equations (9) and (10) can be rewritten as

(13) $${\dot{p}}_{1}=\frac{\beta_{e}}{V_{1}}\left[q_{1}-A_{1}{\dot{x}}_{p}-C_{t}\left(p_{1}-p_{2}\right)\right],$$

(14) $${\dot{p}}_{2}=\frac{\beta_{e}}{V_{2}}\left[-q_{2}+A_{2}{\dot{x}}_{p}+C_{t}\left(p_{1}-p_{2}\right)\right].$$

In hydraulic system modeling and analysis, the piston rod is typically chosen as the focal point of study because the load characteristics significantly impact the dynamic performance of the system’s power components. In this context, various forms of resistance, which are challenging to model precisely, such as inertial load, elastic load, external load, friction, viscous resistance, etc., can be assumed to act upon the hydraulic cylinder. By analyzing the force exerted by the piston rod, the relationship between the force outputted by the hydraulic cylinder and the load force encountered during normal operation can be established. This analysis leads to the derivation of the force balance equation, as demonstrated in Equation (15):

(15) $$p_{1}A_{1}-p_{2}A_{2}=m{\ddot{x}}_{p}+Kx_{p}+F,$$

where m is the equivalent load mass, K is the elastic stiffness of the load, and F is the equivalent load force.

By combining Equations (4), (5) and (13)–(15), the system state variable can be defined as

(16) $$x={\left[x_{1},\;x_{2},\;x_{3},\;x_{4}\right]}^{T}={\left[x_{p},\;{\dot{x}}_{p},\;p_{1},\;p_{2}\right]}^{T}.$$

Then,

(17) $${\dot{x}}_{1}=x_{2}.$$

From (15), it can be demonstrated as

(18) $${\dot{x}}_{2}=\frac{1}{m}\left(A_{1}x_{3}-A_{2}x_{4}-Kx_{1}-F\right).$$

Combining Equations (4), (5), (13) and (14) provides

(19) $${\dot{x}}_{3}=\frac{\beta_{e}}{V_{1}}\left(q_{N}\frac{u}{u_{\max}}\sqrt{\frac{\Delta p_{1}}{\Delta p_{N}}}-A_{1}x_{2}-C_{t}\left(x_{3}-x_{4}\right)\right),$$

(20) $${\dot{x}}_{4}=\frac{\beta_{e}}{V_{2}}\left(-q_{N}\frac{u}{u_{\max}}\sqrt{\frac{\Delta p_{2}}{\Delta p_{N}}}+A_{2}x_{2}+C_{t}\left(x_{3}-x_{4}\right)\right).$$

From Equations (17)–(20), the system state space equation of the electro-hydraulic position servo system can be obtained, as shown in Equation (21):

(21) $$\left\{\begin{array}{l} {\dot{x}}_{1}=x_{2}\\ {\dot{x}}_{2}=\frac{1}{m}\left(A_{1}x_{3}-A_{2}x_{4}-Kx_{1}-F\right)\\ {\dot{x}}_{3}=\frac{\beta_{e}}{V_{1}}\left(B_{N}\sqrt{\Delta p_{1}}u-A_{1}x_{2}-C_{t}\left(x_{3}-x_{4}\right)\right)\\ {\dot{x}}_{4}=\frac{\beta_{e}}{V_{2}}\left(-B_{N}\sqrt{\Delta p_{2}}u+A_{2}x_{2}+C_{t}\left(x_{3}-x_{4}\right)\right)\\ y=x_{1} \end{array}\right.,$$

(22) $$B_{N}=q_{N}\frac{1}{u_{\max}}\sqrt{\frac{1}{\Delta p_{N}}}.$$

The single hydraulic cylinder motion trajectory tracking controller’s performance directly determines the synchronization control’s accuracy. Considering the complexity of the electro-hydraulic position servo system, the tracking accuracy and robustness of the single hydraulic cylinder motion trajectory tracking controller are limited to a certain extent demand. For this reason, the following accuracy of the single cylinder during the design process of the synchronous controller should be considered. Next, the synchronous controller is designed to ensure the single-cylinder following accuracy and double-cylinder synchronization accuracy.

## 3. Adaptive Sliding Mode Synchronous Control (ASMSC)

### 3.1. Co-Simulation AMESim Model of Hoist Synchronization System

**Figure 1:**
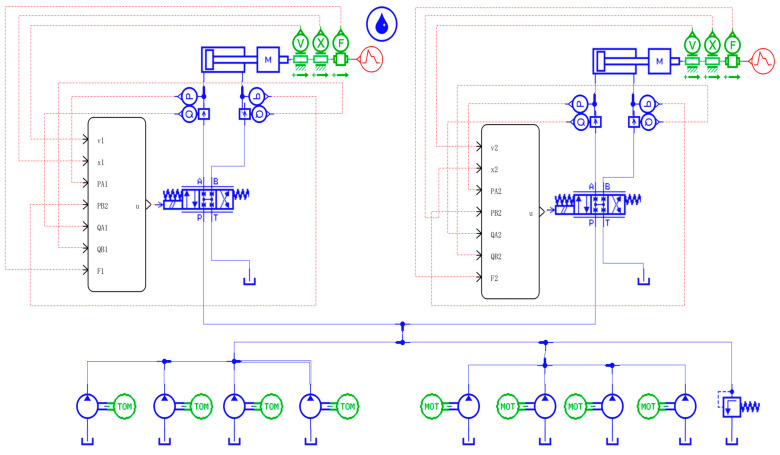
AMESim simulation model diagram of the double-cylinder synchronous system.

According to the hydraulic schematic diagram of the hoist synchronization system designed in Section 2, a model of the AMESim part of the Co-simulation part of the hoist double-cylinder synchronization system is established. As shown in Figure 1, a servo valve is used to control a hydraulic cylinder. Since the simulation is only theoretical research and analysis, slight changes are made to the simulation parameters of different branches to create a displacement difference when the piston rods of the double hydraulic cylinders move.

### 3.2. Synchronous Controller Design

This paper adopts the cross-coupling method in the synchronization control strategy to provide feedback for the synchronization error between the double hydraulic cylinders. In the design of the synchronous controller, the synchronization error between the double hydraulic cylinders is utilized as a compensatory element. The relatively fast-moving hydraulic cylinder’s negative feedback is compensated in that cylinder’s SMC. In contrast, the relatively slow positive feedback of the other hydraulic cylinder is compensated in the sliding mode variable structure controller of that cylinder. When the positive and negative feedback compensation terms of the double hydraulic cylinders are identical, the balance between the double hydraulic cylinders’ synchronization error is not compromised. This approach enables the ASMSC to not only meet the position control accuracy requirements of a single hydraulic cylinder in a double-cylinder electro-hydraulic position servo system but also ensure synchronization stability and robustness.

For the double-cylinder electro-hydraulic position servo control system of the hoist, it is essential to consider not only the system’s ability to track a given signal when a single hydraulic cylinder is in operation but also the accuracy of synchronization between double interlinked hydraulic cylinders. In other words, minimizing the synchronization error between the double hydraulic cylinders is crucial. The position tracking error of a single hydraulic cylinder is defined as

(23) $$h_{i}=x_{ip}-x_{d},$$

where $x_{d}$ is the desired position signal.

The synchronization error between double hydraulic cylinders is defined as

(24) $$\left\{\begin{array}{l} \gamma_{1}=h_{1}-h_{2}\\ \gamma_{2}=h_{2}-h_{1} \end{array}\right.,$$

where $\gamma_{1}$ and $\gamma_{2}$ are the synchronization errors between the double hydraulic cylinders. It can be seen from Equation (24) that to ensure $\gamma_{1}=0$ and $\gamma_{2}=0$, the condition $h_{1}=h_{2}$ must be met. Therefore, there is no synchronization error between the double hydraulic cylinders, so the synchronous displacement of the double hydraulic cylinders can be ensured. In order to more intuitively represent the relationship between the synchronization error between double hydraulic cylinders and the following error of a single hydraulic cylinder, Equation (24) is expressed in matrix form:

(25) γ=TH,

where $\gamma ={\left[\gamma_{1}\gamma_{2}\right]}^{T}$, $H={\left[h_{1}h_{2}\right]}^{T}$, and $T=\left[\begin{array}{ll} 1 & -1\\ -1 & \;\;\;1 \end{array}\right]$.

If H=0, the tracking error and synchronization error converge simultaneously.

The simulation sets the load mass of hydraulic cylinder 2 to be larger than that of hydraulic cylinder 1. For hydraulic cylinder 1, which moves relatively fast, the synchronization error is introduced, and a linear coupled sliding mode surface is designed that includes single-cylinder following error and double-cylinder synchronization error:

(26) $$s_{1}=\left(c_{1}h_{1}+c_{2}{\dot{h}}_{1}+{\ddot{h}}_{1}\right)+\left(\lambda_{1}\gamma_{1}+\lambda_{2}{\dot{\gamma }}_{1}+\lambda_{3}{\ddot{\gamma }}_{1}\right),$$

where $c_{1}$, $c_{2}$, $\lambda_{1}$, $\lambda_{2}$, and $\lambda_{3}$ are all positive real numbers, and their values jointly determine the dynamic quality of the sliding mode.

From Equations (21) and (23), the following can be determined:

(27) $$\begin{array}{ll} {\dddot{h}}_{1} & ={\dddot{x}}_{p}-{\dddot{x}}_{d}\\ & =\frac{1}{m}\left(\begin{array}{l} -Kx_{2}-\frac{A_{1}\beta_{e}}{V_{1}}\left(A_{1}x_{2}+C_{t}\left(x_{3}-x_{4}\right)\right)-\\ \frac{A_{2}\beta_{e}}{V_{2}}\left(A_{2}x_{2}+C_{t}\left(x_{3}-x_{4}\right)\right) \end{array}\right)\\ & +\frac{1}{m}\left(\frac{A_{1}\beta_{e}}{V_{1}}B_{N}\sqrt{\Delta p_{1}}+\frac{A_{2}\beta_{e}}{V_{2}}B_{N}\sqrt{\Delta p_{2}}\right)u_{1}-{\dddot{x}}_{d}-\frac{1}{m}\dot{F} \end{array},$$

where

(28) $$f(x)=\frac{1}{m}\left(-Kx_{2}-\frac{A_{1}\beta_{e}}{V_{1}}\left(A_{1}x_{2}+C_{t}\left(x_{3}-x_{4}\right)\right)-\frac{A_{2}\beta_{e}}{V_{2}}\left(A_{2}x_{2}+C_{t}\left(x_{3}-x_{4}\right)\right)\right),$$

(29) $$g(x)=\frac{1}{m}\left(\frac{A_{1}\beta_{e}}{V_{1}}B_{N}\sqrt{\Delta p_{1}}+\frac{A_{2}\beta_{e}}{V_{2}}B_{N}\sqrt{\Delta p_{2}}\right),$$

(30) $$d_{t}=\frac{1}{m}\dot{F}.$$

The controlled object can achieve operating indicators by designing a suitable controller. The goal is for the motion point on the non-sliding mode switching surface s=0 to move to the sliding mode switching surface within a certain period of time. At the same time, the controller can also achieve the dynamic and static response, control accuracy, and anti-interference ability requirements of the controlled object.

(31) $${\dot{s}}_{1}=0.$$

The approach motion requires a short approach period and as small an arrival rate as possible to reach the switching surface to shorten the approach time without generating a large sliding mode jitter. This article chooses the most commonly used exponential approaching law. For the exponential approaching law, it is enough to determine two parameters for the sliding mode jitter to be weakened. Here, the exponential approaching law is used as the switching control:

(32) $${\dot{s}}_{1}=-\xi \mathrm{sgn}\left(s_{1}\right)-ks_{1},\;k>0,\;\;\xi >0,$$

where sgn(*s*) can be expressed as Equation (33):

(33) $$\mathrm{sgn}\left(s_{1}\right)=\left\{\begin{array}{ll} 1 & s_{1}\geq 0\\ -1\; & s_{1}<0 \end{array}\right..$$

It can be seen that the sign function sgn(*s*) is discontinuous, which will cause chattering in the system, which is not conducive to the system. The continuous hyperbolic tangent function is replaced with the discontinuous sign function to weaken the chattering.

The hyperbolic tangent function is defined as

(34) $$\tanh\left(\frac{x}{\varepsilon }\right)=\frac{e^{\frac{x}{\varepsilon }}-e^{-\frac{x}{\varepsilon }}}{e^{\frac{x}{\varepsilon }}+e^{-\frac{x}{\varepsilon }}},$$

where ε>0 is the value that determines the jitter of the hyperbolic tangent function.

Designing an adaptive approach law including the linear combination of hydraulic cylinder 1 following error and synchronization error, the approach law becomes

(35) $$\left\{\begin{array}{l} {\dot{s}}_{1}=-\xi T\left(s_{1}\right)\tanh\left(\frac{s_{1}}{\varepsilon }\right)-ks_{1}{\left[\tanh\left(\frac{s_{1}}{\varepsilon }\right)\right]}^{2}\\ T\left(s_{1}\right)=\frac{\chi }{\phi {\left[e^{-\left|s_{1}\right|}\right]}^{\varphi }+1/\left|\alpha h_{1}+\beta \gamma_{1}\right|+1} \end{array}\right.,$$

where ξ>0, k>0, χ>0, ϕ>0, φ>0, α>0, β>0, and $T\left(s_{1}\right)>0$. When the system state variable is far away from the sliding mode surface, the system state will exponentially converge to the sliding mode surface. When the system state is close to the sliding mode surface, that is, when $s_{1}$ approaches zero, $T\left(s_{1}\right)$ approaches $\frac{\chi \left|\alpha h_{1}+\beta \gamma_{1}\right|}{(1+\phi )\left|\alpha h_{1}+\beta \gamma_{1}\right|+1}$; further, when the tracking error of hydraulic cylinder 1 $h_{1}$ and the synchronization error of the double cylinders $\gamma_{1}$ approach zero, $T\left(s_{1}\right)$ approaches zero, which can suppress chattering.

When $u_{eq1}$ is the state control item in the designed ASMSC of hydraulic cylinder 1, related to the status of the hydraulic cylinder itself, it can continuously approach ${\dot{s}}_{1}=0$. This kind of control allows for better tracking capabilities of system status.

Combining Equations (26)–(31) and Equation (35), the following can be determined:

(36) $$\begin{array}{ll} {\dot{s}}_{1} & =\left(c_{1}{\dot{h}}_{1}+c_{2}{\ddot{h}}_{1}+{\dddot{h}}_{1}\right)+\left(\lambda_{1}{\dot{\gamma }}_{1}+\lambda_{2}{\ddot{\gamma }}_{1}+\lambda_{3}{\dddot{\gamma }}_{1}\right)\\ & =\left(c_{1}{\dot{h}}_{1}+c_{2}{\ddot{h}}_{1}+f\left(x\right)+g\left(x\right)u_{1}-{\dddot{x}}_{d}-d_{t}\right)+\left(\lambda_{1}{\dot{\gamma }}_{1}+\lambda_{2}{\ddot{\gamma }}_{1}+\lambda_{3}{\dddot{\gamma }}_{1}\right)\\ & =-\xi T\left(s_{1}\right)\tanh\left(\frac{s_{1}}{\varepsilon }\right)-ks{\left[\tanh\left(\frac{s_{1}}{\varepsilon }\right)\right]}^{2} \end{array}.$$

The expression of the synchronization controller is obtained as follows:

(37) $$u_{1}=u_{eq1}+u_{sw1}+u_{r1},$$

where

(38) $$\left\{\begin{array}{l} u_{eq1}=\frac{-c_{1}{\dot{h}}_{1}-c_{2}{\ddot{h}}_{1}-f\left(x\right)+{\dddot{x}}_{d}+d_{t}}{g\left(x\right)}\\ u_{sw1}=\frac{-\xi T\left(s_{1}\right)\tanh\left(\frac{s_{1}}{\varepsilon }\right)-ks{\left[\tanh\left(\frac{s_{1}}{\varepsilon }\right)\right]}^{2}}{g\left(x\right)}\\ u_{r1}=\frac{-\lambda_{1}{\dot{\gamma }}_{1}-\lambda_{2}{\ddot{\gamma }}_{1}-\lambda_{3}{\dddot{\gamma }}_{1}}{g\left(x\right)} \end{array}\right..$$

In Equation (38), $u_{eq1}$ is the state control item in the designed ASMSC of hydraulic cylinder 1, related to the status of the hydraulic cylinder itself; $u_{sw1}$ is a toggle control; $u_{r1}$ is the double-cylinder coupling control compensation term, which is related to the synchronization error between the double hydraulic cylinders.

For hydraulic cylinder 1, in the simulation settings of this chapter, the load of hydraulic cylinder 1 is lighter than the load of hydraulic cylinder 2. When other simulation parameters of the two hydraulic circuits are consistent, hydraulic cylinder 1 runs faster than hydraulic cylinder 2, so $\gamma_{1}>0$. In the previous description, $\lambda_{1}>0$, $\lambda_{2}>0$, and $\lambda_{3}>0$. Therefore, the coupling control compensation term in the hydraulic cylinder 1 synchronization controller is $u_{r}<0$, and it is possible to slow down the relatively fast-moving hydraulic cylinder 1.

For hydraulic cylinder 2, which moves relatively slowly, the coupled sliding mode surface becomes

(39) $$s_{2}=\left(c_{1}h_{2}+c_{2}{\dot{h}}_{2}+{\ddot{h}}_{2}\right)+\left(\lambda_{1}\gamma_{2}+\lambda_{2}{\dot{\gamma }}_{2}+\lambda_{3}{\ddot{\gamma }}_{2}\right).$$

In the same way, the third-order derivative ${\dddot{h}}_{2}$ of the tracking error of hydraulic cylinder 2 is expressed as

(40) $${\dddot{h}}_{2}=f\left(x\right)+g\left(x\right)u_{2}-{\dddot{x}}_{d}-d_{t}.$$

The reaching law is consistent with the previously used exponential reaching law based on the hyperbolic tangent function, based on the combined Equations (35), (39) and (40). The expression of the sliding mode synchronous controller of the hydraulic cylinder 2 is

(41) $$u_{2}=u_{eq2}+u_{sw2}+u_{r2},$$

where

(42) $$\left\{\begin{array}{l} u_{eq2}=\frac{-c_{1}{\dot{h}}_{2}-c_{2}{\ddot{h}}_{2}-f\left(x\right)+{\dddot{x}}_{d}+d_{t}}{g\left(x\right)}\\ u_{sw2}=\frac{-\xi T\left(s_{2}\right)\tanh\left(\frac{s_{2}}{\varepsilon }\right)-ks{\left[\tanh\left(\frac{s_{2}}{\varepsilon }\right)\right]}^{2}}{g\left(x\right)}\\ u_{r2}=\frac{-\lambda_{1}{\dot{\gamma }}_{2}-\lambda_{2}{\ddot{\gamma }}_{2}-\lambda_{3}{\dddot{\gamma }}_{2}}{g\left(x\right)} \end{array}\right..$$

$u_{eq2}$ and $u_{sw}$ are the status control items and switching control items in the ASMSC of hydraulic cylinder 2. It should be noted that for hydraulic cylinder 2, the movement speed is slower than that of hydraulic cylinder 1, so $\gamma_{2}<0$ but $\lambda_{1}>0$, $\lambda_{2}>0$, and g(x)>0 according to Formula (29); then, when $u_{r2}>0$, the coupling control compensation term of hydraulic cylinder 2 is positive. The designed ASMSC can make the originally faster hydraulic cylinder 1 slower and the originally slower hydraulic cylinder 2 faster while ensuring single-cylinder following accuracy. Therefore, in the input of the designed ASMSC, in addition to the trajectory tracking error of a single hydraulic cylinder, it also includes the synchronization error between the double hydraulic cylinders, which is very important for improving the performance of the double hydraulic cylinders. The synchronization accuracy of the cylinders also plays a role.

### 3.3. Synchronous Controller Stability Analysis

Take the ASMSC of hydraulic cylinder 1 for stability analysis. The same is true for hydraulic cylinder 2, and the Lyapunov function is defined as

(43) $$V\;=\frac{1}{2}s_{1}{}^{2}.$$

The simultaneous Equations (36) and (43) can be obtained as follows:

(44) $$\dot{V}=s_{1}{\dot{s}}_{1}=s_{1}\left(-\xi T\left(s_{1}\right)\tanh\left(\frac{s_{1}}{\varepsilon }\right)-ks{\left[\tanh\left(\frac{s_{1}}{\varepsilon }\right)\right]}^{2}\right).$$

**Lemma** **1** ([42])**.*** For any given real number* x*, there exists the following inequality:*

(45) $$x\tanh\left(\frac{x}{\varepsilon }\right)=\left|x\tanh\left(\frac{x}{\varepsilon }\right)\right|=\left|x\right|\left|\tanh\left(\frac{x}{\varepsilon }\right)\right|\geq 0.$$

According to Lemma 1,

(46) $$s_{1}\tanh\left(\frac{s_{1}}{\varepsilon }\right)=\left|s_{1}\right|\left|\tanh\left(\frac{s_{1}}{\varepsilon }\right)\right|\geq 0.$$

In the previous description, ξ, k, and $T\left(s_{1}\right)$ are greater than zero, combining Equations (44) and (46), which are straightforward to show that

(47) $$\begin{array}{ll} {\dot{V}}_{1} & =s_{1}{\dot{s}}_{1}=s_{1}\left(-\xi T\left(s_{1}\right)\tanh\left(\frac{s_{1}}{\varepsilon }\right)-ks_{1}{\left[\tanh\left(\frac{s_{1}}{\varepsilon }\right)\right]}^{2}\right)\\ & =-ks_{1}{}^{2}{\left[\tanh\left(\frac{s_{1}}{\varepsilon }\right)\right]}^{2}-\xi T\left(s_{1}\right)s_{1}\tanh\left(\frac{s_{1}}{\varepsilon }\right)\\ & =-ks_{1}{}^{2}{\left[\tanh\left(\frac{s_{1}}{\varepsilon }\right)\right]}^{2}-\xi T\left(s_{1}\right)\left|s_{1}\right|\left|\tanh\left(\frac{s_{1}}{\varepsilon }\right)\right|\;\leq 0 \end{array}.$$

Therefore, the designed synchronous control system is stable, and the same is true for hydraulic cylinder 2.

## 4. Simulation Analysis

### 4.1. Parallel Synchronization Control

**Figure 2:**
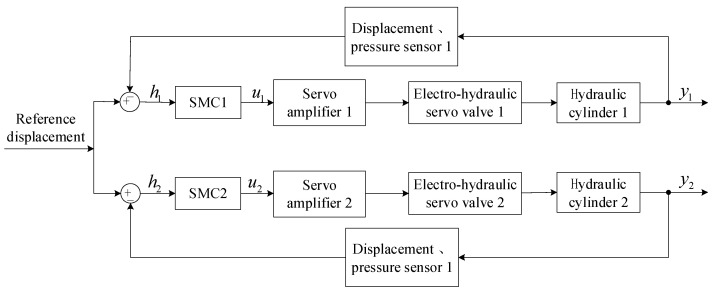
The block diagram of the “parallel synchronous control” system applied to the electro-hydraulic servo system of double hydraulic cylinders.

For the purpose of observing the displacement difference in the double hydraulic cylinders without adding a synchronous controller, let the values of $\lambda_{1}$, $\lambda_{2}$, and $\lambda_{3}$ be 0. The ASMSC designed in this article degenerates into a single hydraulic cylinder trajectory tracking SMC designed to consider the position tracking of a single hydraulic cylinder and will no longer have the relationship between the double hydraulic cylinders. The synchronization error compensation function changes from cross-coupling control to parallel synchronization control. In the AMESim model, the left and right hydraulic cylinders are eccentrically loaded, and the load mass of hydraulic cylinder 2 is increased by 33.3%, while the load of hydraulic cylinder 1 remains unchanged. Figure 2 shows a block diagram of an electro-hydraulic position servo system where “parallel synchronous control” is applied to double hydraulic cylinders. Due to the fact that this control method does not control the synchronization error of the double hydraulic cylinders, although one hydraulic cylinder can work normally, there will definitely be a synchronization error between the double hydraulic cylinders.

**Figure 3:**
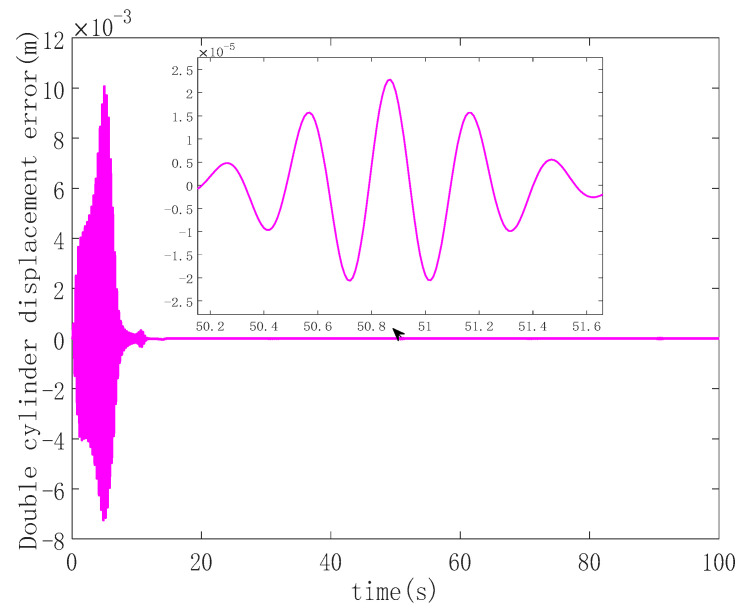
Synchronization error curve when no synchronous control is applied to the double hydraulic cylinders.

Providing a sinusoidal signal, the simulation sets the load mass of hydraulic cylinder 2 to be larger than the load mass of hydraulic cylinder 1. The maximum synchronization deviation of the system and the stability of the system in the parallel synchronous control mode are observed without applying a double-cylinder synchronous controller. The maximum synchronization deviation is achieved in the aforementioned state, and the simulation result is shown in Figure 3.

It can be seen from Figure 3 that for a given sinusoidal signal, for double hydraulic cylinders without a synchronous controller, the maximum synchronization deviation occurs in the initial stage, and the maximum synchronization deviation is approximately 10 mm. After the double hydraulic cylinders reach a steady state, the maximum deviation between the double cylinders is 0.023 mm, and the curve is also sinusoidal.

### 4.2. Simulation Analysis of Double-Cylinder Sliding Mode Cross-Coupling Synchronous Control

**Figure 4:**
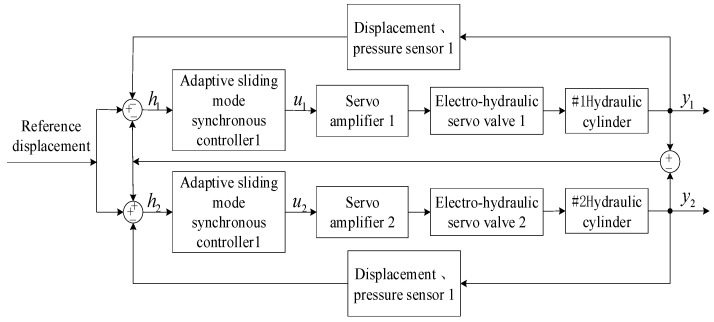
Block diagram of the cross-coupled ASMSC system of the double-cylinder electro-hydraulic position servo system.

To test the control effect of the double-cylinder ASMSC designed in this article, this section will use the double-cylinder electro-hydraulic position servo system as the control object for simulation analysis. To build the Simulink simulation model, the block diagram of the cross-coupled sliding mode synchronous control system of the double-cylinder electro-hydraulic position servo system is provided, as shown in Figure 4.

In the simulation settings, the starting lengths of the two chambers of the hydraulic cylinder in the AMESim model are the same. The system and controller-related parameters in the ASMSC are shown in the Table 1. Among them, $c_{1}$, $c_{2}$, and $\lambda_{1-3}$ are the values obtained through continuous debugging to effectively increase the simulation effect.

**Figure 5:**
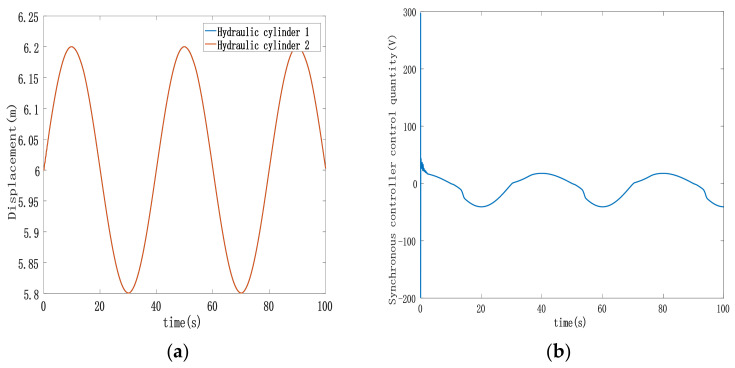
(**a**) Displacement trajectory of double-cylinder under ASMSC; (**b**) control input curve with synchronous controller.

**Figure 6:**
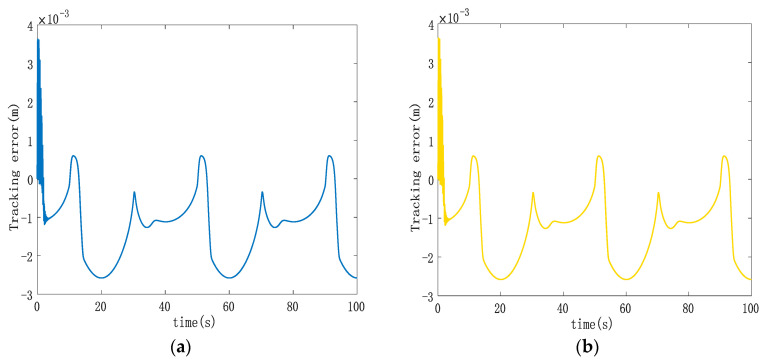
(**a**) Trajectory tracking error curve of hydraulic cylinder 1; (**b**) trajectory tracking error curve of hydraulic cylinder 2.

**Figure 7:**
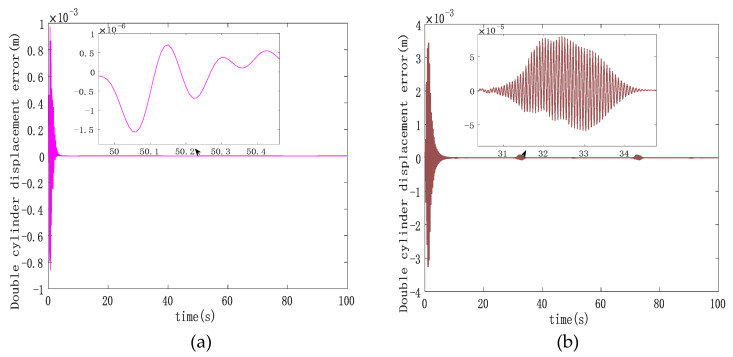
(**a**) Double-cylinder synchronization error curve based on cross-coupled ASMSC; (**b**) double-cylinder synchronization error curve based on cross-coupled PD synchronization control.

Figure 5, Figure 6 and Figure 7 display various aspects of the double-cylinder system, including the displacement and control volume curves when both hydraulic cylinders track the same specified displacement signal and the curve depicting single hydraulic cylinder tracking errors. The synchronization error curves show the double hydraulic cylinders under the ASMSC and the PD synchronous controller (with appropriate values set $K_{p}$ and $K_{i}$ adjusted to 300 and 70, respectively). Figure 5 illustrates that when both hydraulic cylinders track sinusoidal displacement, their displacement tracking curves remain relatively smooth. The actual displacements of the double hydraulic cylinders closely align with the expected signal without any significant hysteresis. Furthermore, the control variable curve generated by the synchronous controller exhibits a smooth output without any noticeable oscillations upon startup.

Analyzing Figure 6, it becomes evident that the double hydraulic cylinders exhibit a significant speed difference during the initial stage due to an unbalanced load. Consequently, each hydraulic cylinder experiences a substantial single-cylinder tracking error in this phase. However, the designed ASMSC effectively mitigates these errors as the system stabilizes. At that point, the maximum errors reach approximately 2.7 mm, with steady-state accuracy less than 1.4% of the given signal amplitude. Figure 7 displays that the maximum synchronization error during system startup is 1 mm. Compared to the system depicted in Figure 3, which lacks a synchronization controller while following the same position signal, the synchronization error at startup is reduced by 90%. In steady-state motion, the maximum synchronization error is merely 0.00152 mm.

In contrast to the maximum steady-state synchronization error of 0.024 mm seen in Figure 3 without a synchronous controller, the designed ASMSC dramatically reduces the maximum synchronization error in the steady state by 93.7%. This improvement is a clear testament to the effectiveness of the synchronization controller, leading to significantly enhanced double-cylinder synchronization accuracy. Both at startup and when the system reaches a steady state, the synchronization accuracy surpasses that of the PD cross-coupled synchronous controller.

### 4.3. Synchronous Controller Robustness Analysis

**Figure 8:**
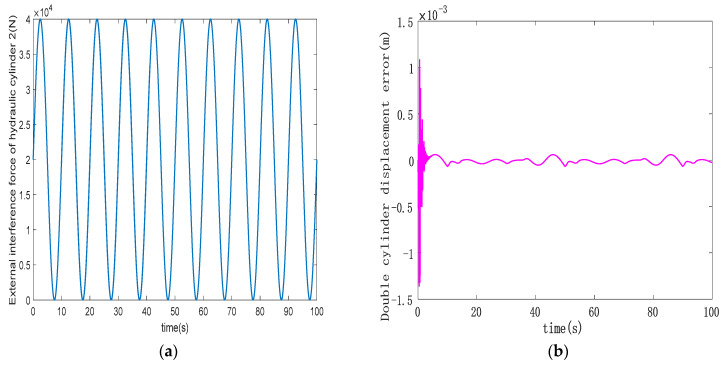
(**a**) Hydraulic cylinder 2 interference force curve; (**b**) double-cylinder synchronization error curve under interference conditions.

**Figure 9:**
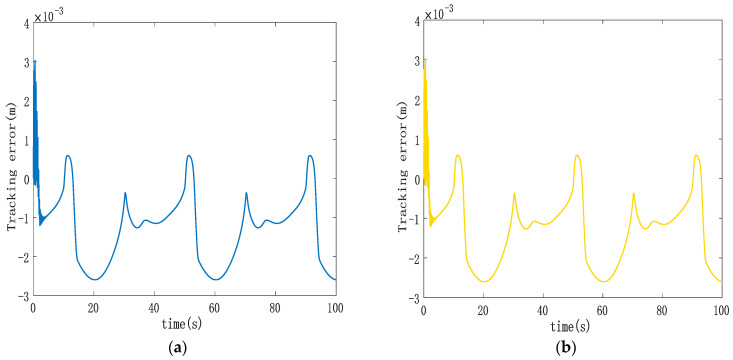
(**a**) Trajectory tracking curve of synchronous control hydraulic cylinder 1 under interference conditions; (**b**) trajectory tracking curve of synchronous control hydraulic cylinder 2 under interference conditions.

To assess the robustness of the designed ASMSC, hydraulic cylinder 1 is not subject to disturbing forces. Instead, a continuously changing sinusoidal disturbance force is subjected to hydraulic cylinder 2, replicating the real-world stresses encountered during the hoist operation. Figure 8 illustrates the disturbance force profile on hydraulic cylinder 2 alongside the synchronization error curve between the double hydraulic cylinders. In Figure 9, the trajectory tracking performance of both hydraulic cylinders is presented. The trajectory tracking errors of the double hydraulic cylinders have not changed significantly compared to previous studies. It can be seen from Figure 8b, compared with Figure 7a, that in the initial stage, the maximum synchronization error between the double hydraulic cylinders increases from 1 mm to 1.35 mm. These findings demonstrate that the external disturbance force applied to hydraulic cylinder 2 does not significantly affect the trajectory tracking performance of an individual hydraulic cylinder or the synchronization between the double hydraulic cylinders. The designed ASMSC possesses a robust anti-interference capability.

## 5. Conclusions

To address the synchronous drive control challenge of the 6300 KN double lifting point hydraulic hoist, this study employed a nonlinear electro-hydraulic position servo system with state variables including piston rod displacement, piston rod speed, rodless chamber pressure, and rod chamber pressure for the asymmetric cylinder. The aim was to create a linear equation of state for control purposes. The focus of the study concerned ASMSC. Traditional SMC can sometimes lead to significant sliding mode jitter due to the presence of sign-switching functions. To mitigate this issue, this paper proposed the use of a continuous hyperbolic tangent function as a replacement for the discontinuous switching function within the exponential approaching law. This modification effectively dampened system jitter. Additionally, the proposed approach combined both the double-cylinder synchronization error variable and the single-cylinder synchronization error variable. It introduced a cylinder following the error variable into the coupling sliding mode surface. This combination aimed to achieve simultaneous convergence of the single cylinder following error and the double cylinder synchronization error. In the method proposed in this article, the integral absolute error of the double-cylinder synchronization was 0.0006963 mm under PD cross-coupling control, and the integral absolute error of the double-cylinder synchronization was 0.004184 mm. Our simulation results indicate that the designed ASMSC outperforms the PD cross-coupled synchronous control method in terms of synchronous control effectiveness.

## Figures and Tables

**Table 1:** Table of electro-hydraulic system and controller-related parameters.

Electro-Hydraulic System and Controller-Related Parameters	Value
m	1.2 × 10^5^ kg
*K*	4 × 10^3^ N/m
$\beta_{e}$	8 × 10^8^ Pa
$A_{1}$	7.85 × 10^−1^ m^2^
$A_{2}$	1.49 × 10^−1^ m^2^
$V_{01}$	4.71 × 10^−3^ m^3^
$V_{02}$	3.59 × 10^−3^ m^3^
$C_{t}$	2 × 10^−11^ m^3^/s·Pa
$B_{N}$	6.074 × 10^−2^ L/(min·V·Pa^1/2^)
$P_{s}$	2.5 × 10^7^ Pa
$P_{0}$	0 Pa
$\Delta P_{N}$	7 × 10^6^ Pa
ε	2 × 10^2^
$c_{1}$	2.55 × 10^6^
$c_{2}$	2.2 × 10^5^
ξ	5
k	2 × 10
χ	1
ϕ	1 × 10^−1^
φ	1 × 10^−1^
α	2 × 10^−1^
β	8 × 10^−1^
$\lambda_{1}$	3 × 10^2^
$\lambda_{2}$	5 × 10^−3^
$\lambda_{3}$	5 × 10^−2^

## Data Availability

Data are contained within the article.
